# Fluid biomarkers across the Alzheimer's disease continuum: assessing their relationship with the patients’ clinical variant and profile

**DOI:** 10.1002/alz70856_102765

**Published:** 2025-12-24

**Authors:** Rosalinda Di Gerlando, Matteo Cotta Ramusino, Francesca Dragoni, Evelyne Minucchi, Bartolo Rizzo, Elisabetta Zardini, Riccardo Rocco Ferrari, Michele Rossi, Giulia Perini, Alfredo Costa, Annalisa Davin, Stella Gagliardi

**Affiliations:** ^1^ Department of Biology and Biotechnology “L. Spallanzani”, University of Pavia, Pavia, Italy; ^2^ Clinical Neuroscience Unit of Dementia, Dementia Research Center, IRCCS Mondino Foundation, Pavia, Italy; ^3^ Molecular Biology and Transcriptomics Unit, IRCCS Mondino Foundation, Pavia, Italy; ^4^ Department of Brain and Behavioral Sciences, University of Pavia, Pavia, Italy; ^5^ Laboratory of Neurobiology and Neurogenetics, Golgi‐Cenci Foundation, Abbiategrasso, Italy; ^6^ Golgi Cenci Foundation, Abbiategrasso, Milan, Italy

## Abstract

**Background:**

Alzheimer's disease (AD) molecular hallmarks are the accumulation of β‐amyloid (Aβ) peptides and phosphorylated tau proteins in the patients’ cerebral cortex. For this reason, the measurement of cerebrospinal fluid (CSF) levels of phosphorylated tau at threonine 181 (pTAU181), Aβ40, and Aβ42 is a routine clinical evaluation that, together with other diagnostic tools, helps providing a correct diagnosis. However, CSF collection is an invasive procedure. Hence, this study aims to compare these biomarkers concentration in CSF and plasma, assess their diagnostic accuracy within AD clinical continuum and determine the clinical variant and profile effect on their concentrations.

**Method:**

Patients were assigned to a specific diagnosis, variant (typical vs atypical AD) and clinical profile (single or multidomain) following the NIA‐AA criteria. Plasma samples were collected from a total of 85 healthy control (HC), 17 mild‐cognitive impairment (MCI non‐AD), 14 MCI presenting positive AD markers (MCI‐AD) and 51 AD patients. CSF was obtained from 10 MCI non‐AD, 12 MCI‐AD and 42 AD dementia cases. *APOE* genotype was assessed using TaqMan™ SNP Genotyping Assay (Applied Biosystems, USA). Both plasma and CSF biomarkers measurements were performed using the LUMIPULSE® G600II instrument (Fujirebio, Japan) with relative kits.

**Result:**

Plasma concentrations of the three biomarkers and relative ratios followed a trend confirmed both by CSF measurements and by literature. Furthermore, both biofluid biomarker levels appeared to change coherently with the change in patients’ MMSE score, further supporting that cognitive function impairment is mirrored by a variation in CSF and most importantly blood biomarker levels. Considering the clinical variant, atypical patients displayed higher pTAU181 and lower Aβ42 concentrations, reflecting their lower MMSE score. As for the clinical profile, the Posterior Cortical Atrophy (PCA) one appeared to deviate from the others, showing higher pTAU181 and lower Aβ42 levels. Lastly, pTAU181 and Aβ42 were observed to be the best AD predictors.

**Conclusion:**

pTAU181, Aβ42, and the Aβ42/Aβ40 ratio were confirmed to be good indicators of AD pathology. Although further analyses are needed to accurately define the role of these biomarkers in distinguishing between the clinical variants, data hints at a possible difference between AD typical and atypical form.

